# An Unusual Ovarian Mucinous Borderline Tumor with a Large Solid Component

**DOI:** 10.1155/2019/1402736

**Published:** 2019-05-22

**Authors:** Eito Kozawa, Kaiji Inoue, Mitsutake Yano, Masanori Yasuda, Kosei Hasegawa, Junji Tanaka, Tomoaki Ichikawa, Mamoru Niitsu

**Affiliations:** ^1^Department of Radiology, Saitama Medical University International Medical Center, 38 Moroyama-chou, Iruma-gun, Saitama 350-0495, Japan; ^2^Department of Pathology Diagnosis, Saitama Medical University International Medical Center, 1397-1 Yamane, Hidaka-City, Saitama 350-1298, Japan; ^3^Departments of Gynecology and Oncology, Saitama Medical University International Medical Center, 1397-1 Yamane, Hidaka-City, Saitama 350-1298, Japan; ^4^Department of Imaging Diagnosis, Saitama Medical University International Medical Center, 1397-1 Yamane, Hidaka-City, Saitama 350-1298, Japan

## Abstract

Herein, we report magnetic resonance imaging (MRI) findings of a mucinous borderline tumor of the ovary, which we observed as a mainly solid tumor with large solid components in the lower pelvic cavity. The appearance of ovarian epithelial tumors on imaging is often complex. Cystic to solid appearing masses may be observed, and they often resemble epithelial carcinoma. Due to mucinous or hemorrhage components of packed small or microcystic components, MRI depicts slightly high signal intensity on T1-weighted images and low signal intensity on T2-weighted images. Mucinous borderline tumor of the ovary with a large solid component is very rare, but it is clinically important to recognize the possibility of mucinous borderline tumor to avoid unnecessary surgical intervention.

## 1. Introduction

Mucinous tumor, a common subtype of epithelial tumor of the ovary, is classified as adenoma, borderline malignancy, or carcinoma based on cytological and structural atypia [1]. In general, magnetic resonance imaging (MRI) detection of a solid component in the tumor suggests primary malignant epithelial tumor of the ovary. MRI findings suggestive of a mucinous borderline malignant tumor include the depiction of a huge multilocular cystic mass with areas of plaque-like thickening in the peritoneal cavity [2–4].

Herein, we describe a case of ovarian mucinous borderline tumor with a large solid component with MRI findings of slightly high signal intensity on T1-weighted imaging and slightly low signal intensity on T2-weighted imaging, which reflected the tumor's histological characteristics.

## 2. Case Report

A 39-year-old woman with no medical history was referred to the Department of Gynecology at our facility after experiencing abdominal pain for the previous 2 weeks. She exhibited no additional symptoms and biological data were normal. Ultrasonography of the pelvis revealed a large mass extending from the right side of the uterine body to the adnexal region. The mass appeared solid and hypoechoic with sound attenuation. Serum levels of carcinoembryonic antigen, carbohydrate antigen 19-9, and carbohydrate antigen 125 were within normal ranges.

**Figure 1:**
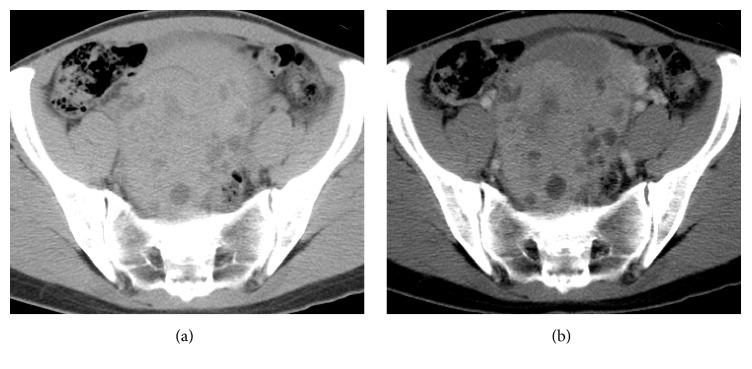
(a) Plain axial computed tomography (CT) depicting a mass of approximately 12 × 9 cm with heterogeneous density. (b) Contrast-enhanced axial CT showing the cystic area of the enhanced solid mass.

**Figure 2:**
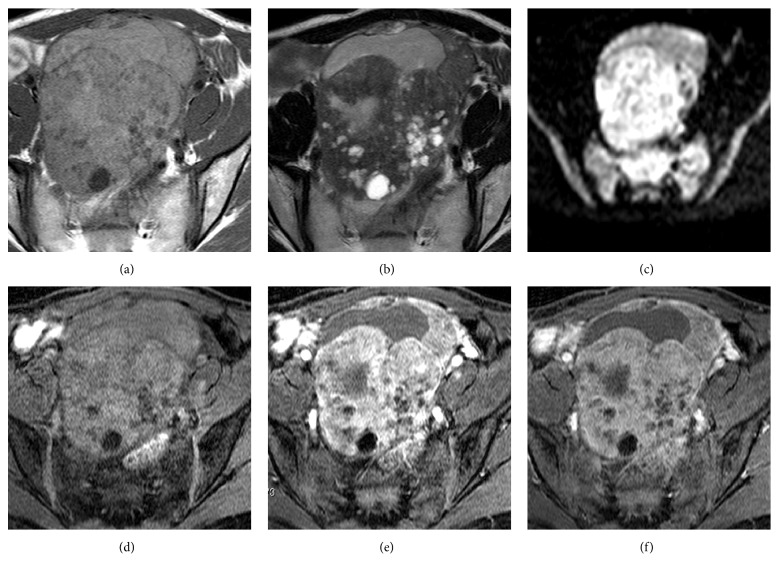
(a) T1-weighted magnetic resonance imaging depicting a mass in the right adnexal region with high signal intensity relative to that of the myometrium. (b) On T2-weighted imaging, the solid component of the mass exhibited slightly low signal intensity, and the large cystic component exhibited high signal intensity. (c) Diffusion-weighted imaging depicting a mass with high signal intensity relative to that of the endometrium. (d) On precontrast fat-saturated T1-weighted imaging, the mass exhibited slightly high signal intensity. (e) On early-phase contrast-enhanced fat-saturated T1-weighted imaging, the mass exhibited strong high signal intensity. (f) On delay-phase contrast-enhanced fat-saturated T1-weighted imaging, the mass exhibited slightly high signal intensity.

The patient then underwent computed tomography (CT) and MRI. Plain CT and contrast-enhanced CT revealed a large solid mass with cystic areas (Figures 1(a) and 1(b)). T1-weighted MRI depicted a mass in the right adnexal region with high signal intensity relative to that of the myometrium (Figure 2(a)). On T2-weighted MRI, the solid component of the mass exhibited low signal that contained small areas of hyperintensity, and the signal intensity of the large cystic component was high (Figure 2(b)). Diffusion-weighted imaging depicted high signal intensity relative to that of the endometrium (Figure 2(c)). In precontrast fat-saturated T1-weighted imaging, the mass exhibited slightly high signal intensity (Figure 2(d)). On early-phase contrast-enhanced fat-saturated T1-weighted imaging, the mass exhibited marked high signal intensity (Figure 2(e)). On delayed-phase contrast-enhanced 3D fat-saturated T1-weighted imaging, the mass exhibited slightly high signal intensity (Figure 2(f)). The preoperative diagnosis was endometrioma with related malignant tumor, such as clear cell carcinoma or endometrioid carcinoma.

**Figure 3:**
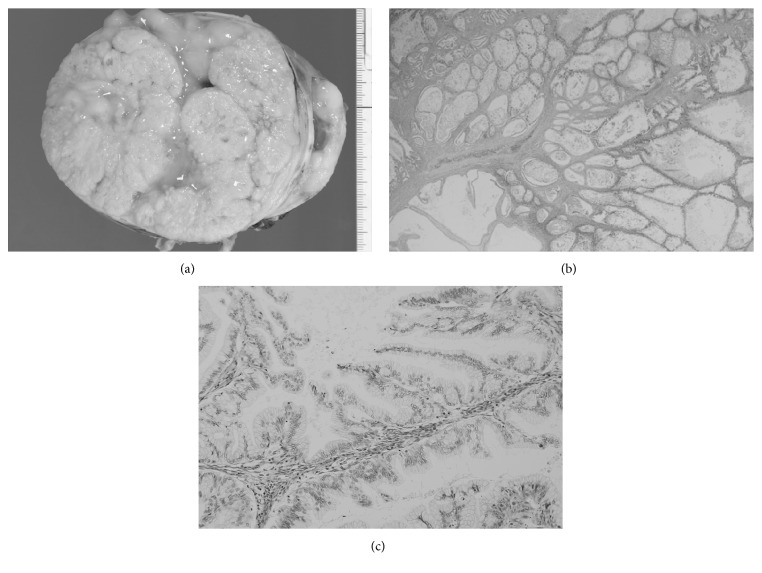
(a) A surgical specimen from right adnexectomy of a 12 × 9 × 7 cm mass revealed a yellowish-white cut surface, a smooth internal surface, and an almost solid component. (b) Microscopy examination revealed multiple small or microcystic spaces that contained mucinous fluid or hemorrhage and ovarian stromal intervening fibrous tissues and multiple vascular spaces. (c) Microscopic examination of the papillary-structured architecture revealed mucus-producing tumor cells with moderate atypia.

The surgical specimen from right adnexectomy consisted of a 12 × 9 × 7 cm mass with a yellowish-white cut surface, a cystic component containing dark yellow fluid, a smooth internal surface, and an almost solid component (Figure 3(a)). Microscopy examination revealed multiple small cystic spaces that contained mucinous fluid or hemorrhage and ovarian stromal intervening fibrous tissues and multiple vascular spaces(Figure 3(b)). Mucus-producing tumor cells with moderate atypia were detected in the papillary-structured architecture. (Figure 3(c)). Closely packed small cysts and microcysts densely filled with mucinous fluid or hemorrhage resembled solid components. On the basis of microscopic examination of a lot of H&E sections, which were prepared to detect malignancy, the tumor was finally diagnosed as an ovarian mucinous borderline tumor of gastrointestinal type. Recovery was uneventful and the patient was discharged 7 days after surgery. No local or systemic recurrence has been detected in the 4 years after the surgery.

## 3. Discussion

In 1973, Hart and Norris [5] first described mucinous borderline tumor as a separate category of mucinous cystadenocarcinoma with multilocular neoplasm and papillary infoldings that do not invade the stroma. Although the prognosis of mucinous carcinoma is poor, that of mucinous borderline tumor is good. Nevertheless, patients with borderline ovarian tumors require long-term follow-up and evaluation because the tumor can reportedly recur up to 20 years after the initial diagnosis [6]. Careful follow-up of these patients via pelvic MRI may be critical to monitor disease recurrence or progression.

The MRI features of mucinous ovarian borderline tumor include a larger size than mucinous cystadenoma and manifestation as a multilocular mass with thick septations and a solid component or components [2–4]. The signal intensity of the loculi varies on both T1-weighted and T2-weighted images (a so-called “stained glass” appearance) depending on the viscosity of the contents, which can include mucin, blood products, and/or debris [2–4]. A solid component, thick septa, and a thick and irregular wall suggest a malignant epithelial ovarian tumor [2–4]. Although a large solid component of the mucinous borderline tumor is very rare, its recognition and correct diagnosis are important for determining the degree of preservation during the ensuing operation, especially in women who wish to become pregnant.

The diffuse proliferation of tumor cells tends to result in malignant lesions exhibiting a proportionately greater solid tissue component [1]. Early phase enhancement of solid components usually suggests a malignant ovarian tumor [7]. In the present case, enhanced solid components contained ovarian stroma with many vascular spaces, so although the solid entities were closely packed with many cystic components, they exhibited strong enhancement.

Yon et al. [8] recently described MRI features for differentiating between borderline and malignant epithelial ovarian tumors. The features of borderline tumor were round or oval shape with well-defined margins and clear cystic-solid interfaces, purely cystic or predominantly cystic with papillae or nodules, branching or exophytic papillae, and the presence of an ipsilateral ovary. The current case only exhibited one of these, the presence of an ipsilateral ovary. Usually the solid component of a malignant tumor exhibits low signal or isosignal intensity compared to myometrium on T1-weighted imaging; however, the present case exhibited slightly high signal intensity. Because pathologically the solid components of MRI scans reflected mucinous fluid and hemorrhage in the packed small cystic components, slightly high signal intensity on T1-weighted imaging may be characteristic.

In summary, we visualized a mucinous borderline tumor of the ovary as a large solid component with slightly high signal intensity on T1-weighted imaging, low signal intensity on T2-weighted imaging, and high signal intensity on diffusion-weighted imaging. These signal patterns reflected fluids of mucinous composition or hemorrhage, and the solid entities were packed cystic components.
